# Effects of posterior staphyloma on choroidal structure in myopic adults: a retrospective study

**DOI:** 10.1186/s12886-023-03158-y

**Published:** 2023-10-09

**Authors:** Zhuo-hua Zhou, Pian-pian Xiong, Jiao Sun, Yan-ling Wang, Jia-lin Wang

**Affiliations:** Department of Ophthalmology, Beijing Friendship Hospital, Capital Medical University, Yong An Road 95th, Tian Qiao Street, Beijing, 100050 China

**Keywords:** Choroidal vascularity index, Posterior staphyloma, Myopia, Axial growth

## Abstract

**Background:**

Studies on the choroid of myopic eyes with posterior staphyloma have shown that choroidal thickness decreased. This retrospective study further analysed the effects of posterior scleral staphyloma on choroidal blood vessels and matrix components compared to non-pathological myopia.

**Methods:**

In this cross-sectional study, ninety-one eyes were divided into pathological (posterior staphyloma) and non-pathological myopia. The latter was further divided into three groups (Group 1: 26 mm ≤ axial length; Group 2: 24 mm ≤ axial length < 26 mm; Group 3: 22 mm ≤ axial length < 24 mm). Choroidal thickness, total choroidal area, luminal area, stromal area, and choroidal vascularity index were calculated.

**Results:**

The CVI in N1, N2, I1, S2 of the posterior staphyloma group were lower than those of group 1 (both *P* < 0.05). The mean height of posterior staphyloma was associated with mean CT (Pearson correlation: *r* = -0.578, *P* = 0.039) but not with the mean CVI in posterior staphyloma group. In all groups, the mean choroidal thickness, total choroidal area, luminal area, and stromal area were significantly associated with axial length (*P* < 0.001), and the mean choroidal vascularity index was significantly associated with the mean choroidal thickness (*P* < 0.001).

**Conclusion:**

The choroidal structure of pathological myopia with posterior staphyloma and non-pathological myopia with longer axial length demonstrates alterations in which choroidal vessels are more impaired than the stroma. A lower choroidal vascularity index should be alert to pathological changes for myopia with axial length > 26 mm.

## Introduction

Myopia has become a globally prevalent ocular disease, that affects patients’ quality of life [1]. Currently, 23% of the population suffers from myopia worldwide, and this percentage is predicted to reach 49.8% by 2050 [2]. Posterior staphyloma (PS) is one of the structural changes of pathological myopia and the principal factor affecting subfoveal choroidal thickness (CT) and volume in patients with high myopia [3, 4]. The choroid plays a vital role in myopia, and its changes tend to occur earlier than those of the retina and long-term eye growth variation [5].

As the refractive state changes, changes in the choroid accordingly alter the position of the retina, changing the focus of the light path to make the image clearer [6]. Animal studies have shown that CT increases in the direction of compensatory defocus in the eyes of animals with simulated myopia [7]. Abnormal axial growth is an intrinsic characteristic of myopia, accompanied by ocular biomechanical changes that result in chorioretinal atrophy, thinning, and other complications [8].

Advances in imaging equipment and measurement techniques have made it possible to quantify choroidal features [9]. CT decreases with myopia progression and is significantly correlated with AL [7, 10]. However, CT is susceptible to many factors, such as age, sex, blood pressure, and spherical equivalent, and it cannot describe detailed changes in choroidal composition [11]. The choroidal vascularity index (CVI) is a more accurate and stable indicator than CT. It refers to the ratio of the luminal area to the total choroidal area and can noninvasively quantify the proportion of nearly all choroidal vessels and perfusion state [12]. Moreover, it has good repeatability and low variability and is not easily affected by other physiological factors [13]. Enhanced depth optical coherence tomography is a practical instrument that can improve choroidal visibility by increasing the signal-to-noise ratio and facilitating CVI calculations [5, 12]. CVI can be used to evaluate the overall choroidal vascular status including medium and large vessels compared with optical coherence tomographic angiography, which is limited by scanning depth and cannot properly display the vessels deep in the choroid and assess the overall vascular status of the choroid [12, 14]. Furthermore, CVI can quantify the status of choroidal vessels and blood perfusion, which can provide more information about choroid than ICGA [12].

Thinning of the choroid in eyes with PS mainly appears in the small vascular layer, followed by the medium blood vessel [15]. The subfoveal and parafoveal choroidal capillary perfusion areas in eyes with PS are smaller than those in no posterior staphyloma eyes [16]. Moreover, these changes may be involved in the formation of neovascularization and myopic maculopathy in eyes with PS [17, 18]. Thus, it is vital to elucidate the effect of PS on the choroidal structure. However, alterations in CVI in eyes with PS have not been reported. Axial length is also a risk factor for myopic maculopathy. It may be positively correlated with the possibility of myopic maculopathy, and the critical limits of axial length for detecting myopic maculopathy are 25.9 mm for men and 25.3 mm for women [19]. Therefore, analysing the relationship between the fundus structure and axial length is essential.

Compared with other young populations grouped by axial length in myopic CVI studies, the inclusion of participants aged 23–48 years without myopic maculopathy in this study [20, 21]. This facilitates early detection of changes in the choroidal structure in myopia because choroidal thinning was significantly faster in highly myopic people aged 20–39 years, and the rate slowed with age [22]. Once over 45 to 50 years of age, the incidence of various macular lesions increases significantly [23].

This study aimed to preliminarily evaluate whether the presence of PS affects choroidal structure using CVI. Furthermore, we further divided nonpathological myopia into three groups according to axial length due to the changes in dioptres and the alterations in spherical equivalent in certain pathological conditions do not exhibit a strictly linear relationship with axial growth, and analysed changes in the choroidal structure to clarify the exact changes in the choroidal vasculature and stroma during axial extension [24].

## Methods

### Participants and examination

A total of 91 adults were included in the study. The inclusion criteria were as follows: (1) age 20–50 years, (2) axial length ≥ 22 mm, (3) best-corrected visual acuity ≥ 1.0, (4) no myopic maculopathy and optic neuropathy, and (5) a clear peripheral fundus. Exclusion criteria were: (1) patients who could not undergo or cooperate with the examination or obtain clear results or the results could not be analysed; (2) intraocular pressure > 21 mmHg; (3) refractive media opacity or abnormalities affecting the examination; and (4) history of endophthalmitis and fundus diseases, other systemic diseases. Finally, the participants were divided into pathological (posterior staphyloma group) and nonpathological myopia. The latter was further divided into three groups: group 1(26 mm ≤ axial length), group 2 (24 mm ≤ axial length < 26 mm), and group 3 (22 mm ≤ axial length < 24 mm).

General information, including age, sex, and blood pressure, was collected. All patients underwent ophthalmologic examinations by a trained optometrist, including anterior segment examination with slit-lamp, intraocular pressure, refractometry performed by an autorefractor (VISUREF 100; Carl Zeiss, Germany), and best-corrected visual acuity using international standard visual acuity chart, which was converted to the minimum resolution logarithm (Log MAR) visual acuity for analysis after measurement. Axial length was measured five times from the apex of the cornea to the retinal pigment epithelium using an optical biometer (IOL Master, Zeiss, Germany), the average value was adopted in the final analysis. Fundus photography by VISUCAM 200 (Zeiss, Germany), and choroidal images using SPECTRALIS® (Heidelberg Engineering GmbH, Heidelberg, Germany) spectral-domain OCT.

### Enhanced depth optical coherence tomography image acquisition

The B-scans of each image used in our study had a quality index of 25 dB, and 100 B-scans were averaged to improve the signal-to-noise ratio. The same retinal scan locations were utilized by setting the follow-up mode in the instrument, and a single line scan of 30° (9 mm) through the centre of the fovea was applied. Horizontal and vertical sections passing through the centre of the fovea were selected for analysis. The image was considered acceptable and used for analysis after two graders determined that the choroid image and boundary were clear and identifiable. Only the right eye was included in the analysis because of interocular correlation.

Considering that the magnification of OCT images is different due to AL elongation, Bennett’s formula is applied for magnification correction to minimize image distortion caused by errors [25].

### Measurement of choroidal parameters

**Fig. 1:**
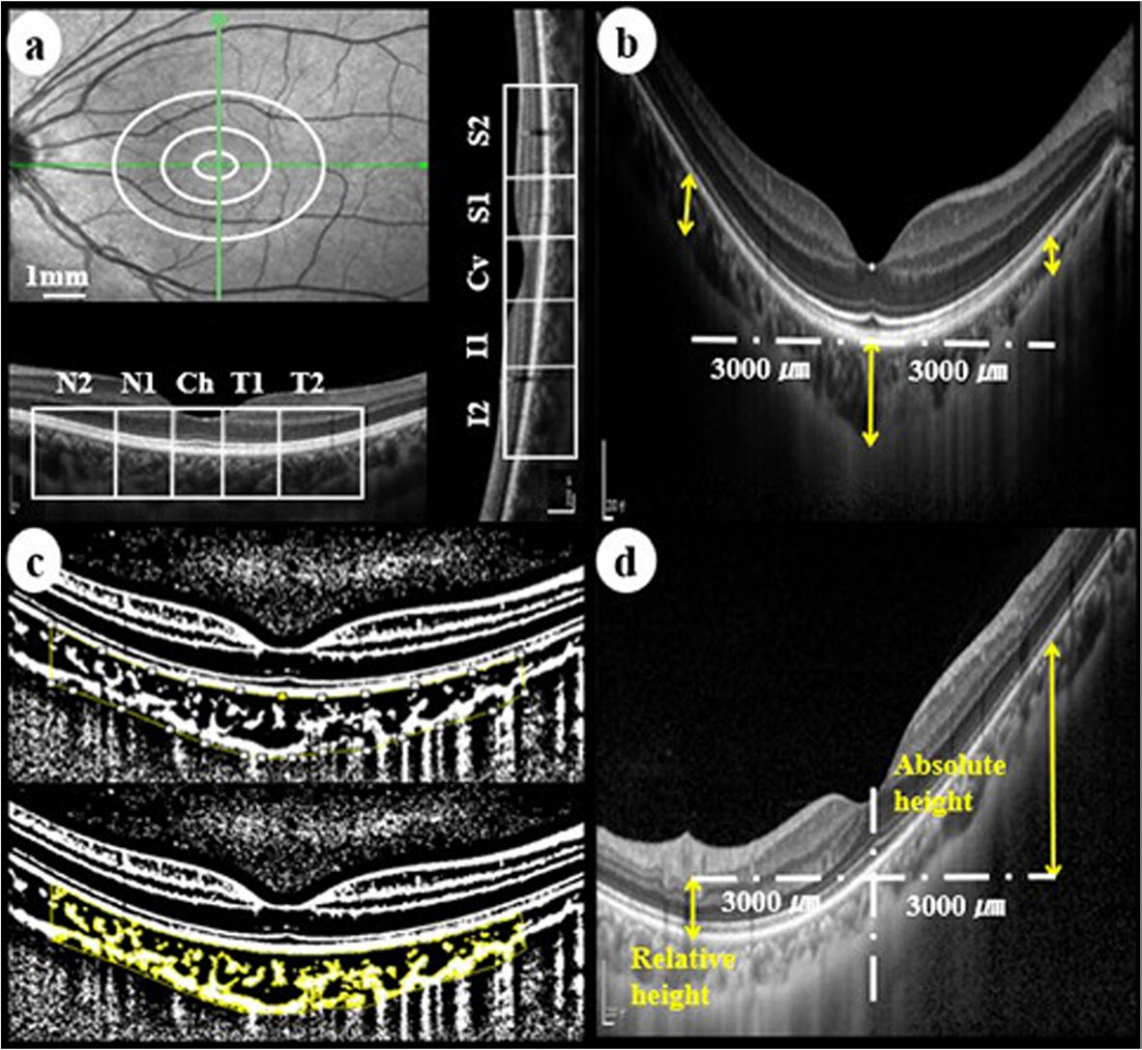
Illustration of macular portion and choroidal parameters measurement. **a** Macular Early Treatment of Diabetic Retinopathy grid and subdivision in horizontal and vertical scans. Quadrants: Ch, central of the fovea horizontally; N1, nasal parafovea; N2, nasal perifovea; T1, temporal parafovea; T2, temporal perifovea; Cv, central of the fovea vertically; I1, inferior parafovea; I2, inferior perifovea; S1, superior parafovea; S2, superior perifovea. **b** Measurement of the choroidal thickness. **c** Image binarization and ROI selection. **d** Measurement of posterior scleral staphyloma height

Based on the Early Treatment of Diabetic Retinopathy grid, the macular region was divided into three concentric circles with diameters of 1 mm (central fovea, area C), 3 mm (parafovea, denoted by 1), and 6 mm (perifovea, denoted by 2) (Shown in Fig. 1a). CT was measured from the outer layer of the retinal pigment epithelium to the choroidal–scleral interface, using the linear measurement tool of OCT at the centre of the fovea and 3 mm nasal, temporal, superior, and inferior from the fovea (shown in Fig. 1b). The average was used as the mean CT. The presence of PS was defined as the curvature of the retinal pigment epithelium layer with a foveal depth ≥ 500 μm relative to the 3 mm periphery of the fovea [26]. In addition, all participants underwent a fundus examination to further determine the results of OCT. The height of the PS was measured as described by Ikuno et al. [27]. The peripheral retinal pigment epithelium located behind the fovea was defined as the absolute height and was denoted as a positive value. Conversely, the relative height was negative (shown in Fig. 1d). The average of these values was recorded as the mean height of the PS.

The ImageJ software version 1.52 (National Institutes of Health, Bethesda, MD, USA) was used for image processing. The image was first converted to 8-bit and binarized using Niblack’s auto local threshold tool to present a clear choroidal–scleral interface, making the selection of the ROI more accurate (shown in Fig. 1c). Finally, the total choroidal area, luminal area, stromal area, and CVI were measured and calculated. The mean CVI was calculated as the average in the four quadrants, 3 mm from the fovea. The mean luminal thickness was defined as the mean CT multiplied by the mean CVI, and stromal thickness was defined as the mean CT minus the mean luminal thickness. Nine images (10%) were analysed by two graders to evaluate the intra and interobserver measurement agreements. After obtaining a good agreement, all images were analysed by a grader.

### Statistical analysis

IBM SPSS22.0 (IBM, Armonk, NY, USA) was used for the statistical analyses. Qualitative analysis of count data using χ^2^ test. The Shapiro–Wilk test was used to test the normality of the measurement data, and a single-factor analysis of variance or independent *t*-test was used to test the normality and homogeneity of variance. Otherwise, non-parametric tests were used. Normal data are expressed as mean ± standard deviation, otherwise represented by median (interquartile range [IQR]). The Intraclass Correlation Coefficient was used to analyse the consistency of measurement. Pearson’s or Spearman’s correlation analysis was used to calculate the degree of correlation between variables and statistical significance. The relationship between the parameters was analysed using linear regression. The difference was considered statistically significant when the two-sided *P* value was less than 0.05.

## Results

### General information

Ninety-one eyes from 91 participants (30 males and 61 females) were included in this study. Their ages ranged from 23 to 48 years, with a median (IQR) age of 35 (13) years. The basic characteristics of the participants in each group were presented in Table 1. There were no significant differences in age, sex, heart rate, systolic blood pressure, diastolic blood pressure, intraocular pressure, spherical equivalent dioptres and best-corrected visual acuity among the four groups (all *P* > 0.05). The repeatability of manual measurement was good with the Intraclass Correlation Coefficient values varying from 0.807 to 0.991 for intraobserver agreement and 0.813 to 0.994 for interobserver agreement.

**Table 1:** Basic characteristics of PS group, Group 1, Group 2 and Group 3

Characteristics	PS group	Group 1	Group 2	Group 3	*P* Value^*^	*P1* Value^†^	*P2* Value^‡^
Eyes, n	13	23	35	20	—	—	—
Age (y)	30.38 ± 5.69	34(13)	37 (15)	36.45 ± 5.61	0.063	0.109	0.507
Sex (M / F), n	9/14	2/11	12/23	7/13	0.523	0.267	0.927
SBP (mmHg)	110.08 ± 5.07	110 (12)	111.00 ± 8.46	107.90 ± 8.30	0.300	0.606	0.189
DBP (mmHg)	76.77 ± 4.82	73.35 ± 8.10	73 (10)	70.90 ± 8.40	0.087	0.175	0.272
Heart rate	79.77 ± 7.73	76.43 ± 6.34	76 (8)	75.40 ± 7.54	0.159	0.170	0.592
SE (dioptres)	-8.61 ± 1.17	-7.89 ± 2.41	-4.04 ± 2.30	-0.63 ± 0.68	< 0.001	0.324	< 0.001
BCVA (Log MAR)	.00 (.00)	.00 (.00)	.00 (.00)	.00 (.00)	0.324	0.537	0.493
IOP (mmHg)	16.02 ± 2.23	15.93 ± 2.55	15.01 ± 2.76	15.18 ± 1.58	0.405	0.917	0.398
AL (mm)	27.06 ± 0.65	26.89 ± 0.69	24.98 ± 0.56	23.23 ± 0.47	< 0.001	0.466	< 0.001

*PS* posterior staphyloma, *M* male, *F* female, *SBP* systolic blood pressure, *DBP* diastolic blood pressure, *SE* spherical equivalent, *BCVA* best-corrected visual acuity, *IOP* intraocular pressure, *AL* axial length

^*^P Value: analysis among the four groups

^†^*P1* value: analysis between PS group and Group 1

^‡^*P2* value: analysis among Group 1, Group 2 and Group 3

### Analysis of choroidal morphological characteristics in groups

**Fig. 2:**
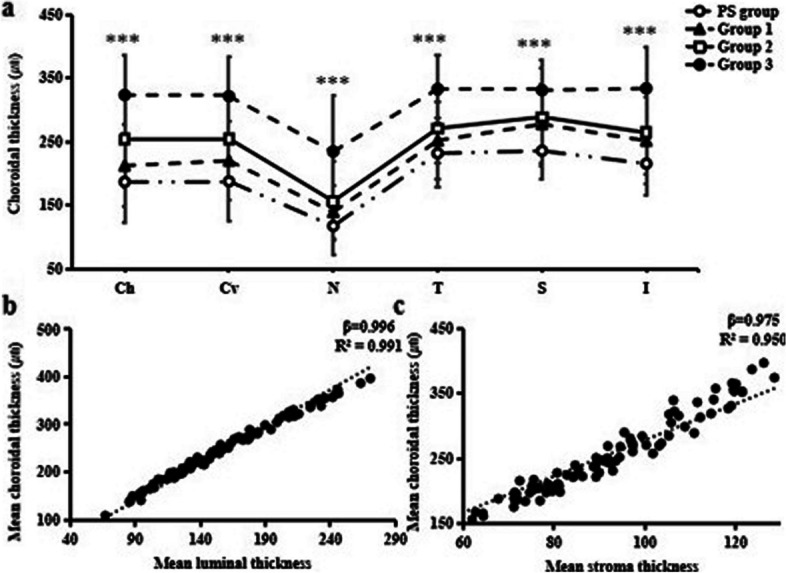
Choroidal thickness tomography among four groups. **a** Choroidal thickness at each quadrant. Ch, central of subfovea horizontally; Cv, central of subfovea vertically; N, nasal; T, temporal; S, superior; I, inferior. **b** Linear analysis of mean luminal thickness and choroidal thickness, **c** Linear analysis of mean stromal thickness and choroidal thickness. *P < 0.05, **P < 0.01 and ***P < 0.001 delegate significant difference among the four groups

The CT in each sector demonstrated significant differences among the four groups (*P* < 0.001) (shown in Fig. 2a). Compared with group 3, the mean CT in PS group, group 1 and group 2 were all significantly decreased (*P* < 0.001). The luminal thickness (R^2^ = 0.991, β = 0.996, *P* < 0.001) significantly decreased compared to stromal thickness (R^2^ = 0.950, β = 0.975, *P* < 0.001) in all groups (shown in Fig. 2b, c). In three non-pathological groups, CT was the thinnest in the nasal sector (*P* < 0.001). Linear regression analysis showed that the horizontally subfoveal CT (R^2^ = 0.295, β =  − 0.550, *P* < 0.001) showed the maximum reduction with axial growth, followed by vertically subfoveal (R^2^ = 0.293, β =  − 0.548, *P* < 0.001) and nasal CT (R^2^ = 0.246, β =  − 0.504, *P* < 0.001).

**Fig. 3:**
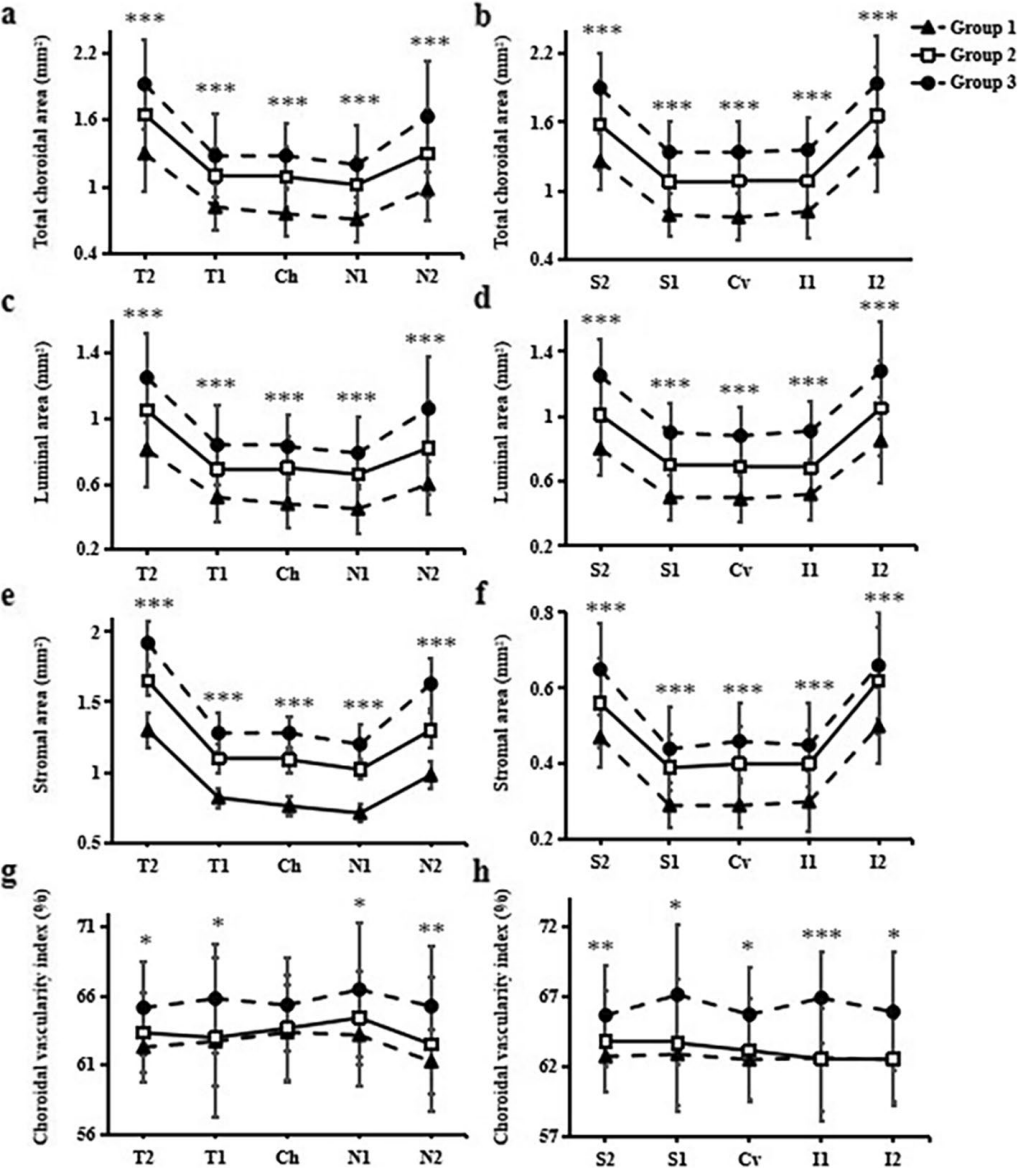
Choroidal vascular distribution among three groups. **a**, **b** Total choroidal area at each sector, **c**, **d** Luminal area at each sector, **e**, **f** Stromal area at each sector, and **g**, **h** Choroidal vascularity index at each sector. Ch, central of the fovea horizontally; N1, nasal parafovea; N2, nasal perifovea; T1, temporal parafovea; T2, temporal perifovea; Cv, central of the fovea vertically; I1, inferior parafovea; I2, inferior perifovea; S1, superior parafovea; S2, superior perifovea. *P < 0.05, **P < 0.01 and ***P < 0.001 delegate significant difference among the three groups

Total choroidal area (shown in Fig. 3a, b), luminal area (shown in Fig. 3c, d), and stromal area (shown in Fig. 3e, f) were significantly different among the three groups (*P* < 0.001). The CVI in the horizontal central fovea was not statistically different among the three groups but showed a tendency to decrease (*P* > 0.05), and decreased in the longer AL group in the remaining sectors (all *P* < 0.05) (shown in Fig. 3g, h). The mean CVI was significantly different among the three groups (*P* < 0.001) (Table 2).

**Table 2:** The CVI and CT measurement results in all groups

Parameter	Region		PS group	Group 1	Group 2	Group 3	*P* Value^*^
CVI (%)		Mean	61.69 ± 2.32	62.71 ± 1.90	63.12 ± 2.20	65.39 ± 2.04	< 0.001
	central fovea	Horizontal	60.99 ± 3.27	63.37 ± 3.44	63.67 ± 3.88	65.35 ± 3.41	0.011
		Vertical	61.82 ± 4.05	62.51 ± 2.88	63.16 ± 3.71	65.72 ± 3.41	0.007
	parafovea	Nasal	60.39 ± 2.92	63.17 ± 3.67	64.42 ± 3.36	66.47 (65.06)	< 0.001
		Temporal	60.65 ± 2.62	62.71 ± 3.21	62.99 (63.79)	65.81 (65.36)	0.001
		Superior	62.05 ± 1.36	62.90 ± 4.06	63.70 (63.37)	67.14 ± 5.03	0.002
		Inferior	59.74 ± 2.89	62.65 (62.07)	62.55 ± 3.67	66.91 ± 3.28	< 0.001
	perifovea	Nasal	58.66 ± 3.04	61.25 ± 2.34	62.50 ± 4.85	65.26 ± 4.34	< 0.001
		Temporal	61.07 (61.31)	62.30 ± 2.48	63.32 ± 2.86	65.15 ± 3.38	0.002
		Superior	60.92 ± 1.54	62.74 ± 2.60	63.82 ± 3.65	65.67 ± 3.60	< 0.001
		Inferior	62.27 ± 2.04	62.51 ± 2.99	62.58 ± 3.36	65.91 ± 4.24	0.002
CT (μm)		Mean	195.72 ± 45.03	225.37 ± 52.84	248.00 ± 59.72	312.73 ± 49.54	< 0.001
	central fovea	horizontal	186.69 ± 63.73	212.22 ± 64.15	253.47 (235)	323.30 ± 61.82	< 0.001
		Vertical	187.46 ± 62.39	220.04 ± 62.80	254.59 ± 71.17	321.28 ± 61.34	< 0.001
	perifovea	Nasal	117.46 ± 44.50	139.30 ± 41.31	156.31 (143)	234.50 ± 89.20	< 0.001
		Temporal	231.77 ± 54.39	251.78 ± 61.66	270.80 ± 53.92	332.75 ± 52.72	< 0.001
		Superior	235.54 (232)	277.52 ± 62.25	288.71 ± 78.00	330.85 ± 46.45	< 0.001
		Inferior	215.38 ± 48.47	251.35 ± 68.82	264.03 ± 64.52	333.65 ± 64.30	< 0.001

*CT* choroidal thickness, *CVI* choroidal vascularity index

^*^P Value: analysis among the four groups

### Analysis of choroidal morphological characteristics of PS Group

**Fig. 4:**
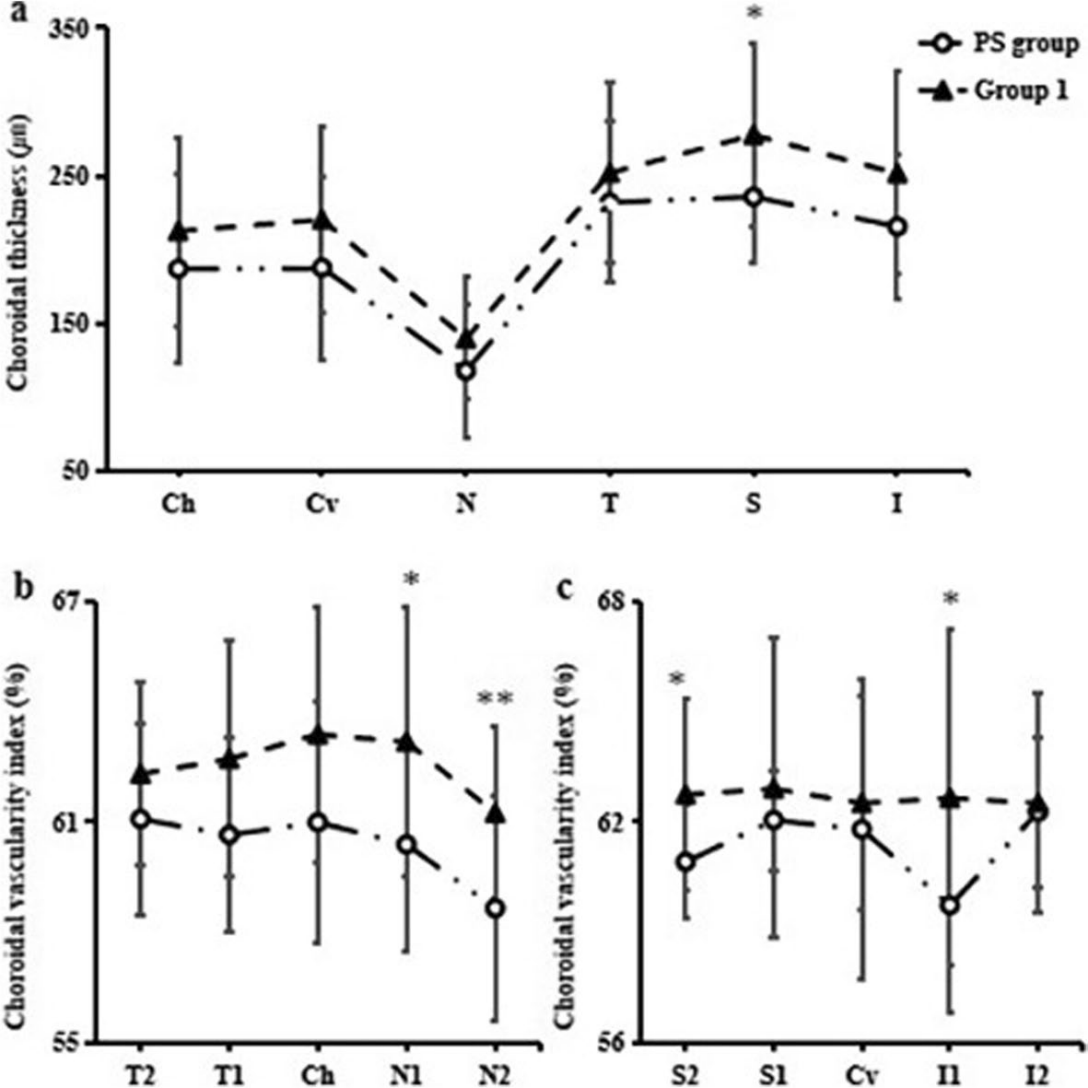
Choroidal morphology characteristics between PS and Group 1. **a** Choroidal thickness at each quadrant. Ch, central of subfovea horizontally; Cv, central of subfovea vertically; N, nasal; T, temporal; S, superior; I, inferior. **b** Choroidal vascular index at each sector. Ch, central of the fovea horizontally; N1, nasal parafovea; N2, nasal perifovea; T1, temporal parafovea; T2, temporal perifovea; Cv, central of the fovea vertically; I1, inferior parafovea; I2, inferior perifovea; S1, superior parafovea; S2, superior perifovea. *P < 0.05, **P < 0.01 and ***P < 0.001 delegate significant difference between the two groups

CT was also the thinnest nasally in the PS group (shown in Fig. 2a). However, compared with group 1, CT in the superior quadrant was significantly different in the PS group (*P* = 0.022), and CT in other quadrants in the PS group was not statistically different from that in group 1, but it tended to become thinner (Fig. 4a). The mean total choroidal area (4.11 ± 0.74 mm^2^ vs 4.79 ± 1.16 mm^2^, *P* = 0.063), luminal area (2.53 ± 0.47 mm^2^ vs 3.02 ± 0.78 mm^2^, *P* = 0.052), stromal area (1.57 ± 0.29 mm^2^ vs 1.78 ± 0.39 mm^2^, *P* = 0.106) and CVI (61.69 ± 2.32 mm^2^ vs 62.71 ± 1.90 mm^2^, *P* = 0.164) were not statistically different in PS group from group 1 but showed a tendency to decrease. The CVI in N1, N2, I1 and S2 of the PS group were decreased than those of group 1 (all *P* < 0.05) (Fig. 4b, c).

PS height was 130.77 ± 214.09 μm nasally, 258.15 ± 193.01 μm temporally, 462.62 ± 142.66 μm superiorly, 32.69 ± 174.43 μm inferiorly. One-way analysis of variance showed a significant difference in the PS height among the four quadrants (*P* < 0.001). The height of the superior quadrant was significantly greater than that of other quadrants (nasal and inferior: both *P* < 0.001; temporal: *P* = 0.039). There was a statistically significant difference between inferior and temporal PS heights (*P* = 0.017).

Table 2 summarised the CVI and CT measurement results in all groups.

### Correlation and regression analysis between parameters

Correlation analysis and univariate linear regression showed that only axial length was significantly associated with the mean CT (Pearson correlation: *r* =  − 0.552, *P* < 0.001; univariate linear regression: R^2^ = 0.297, β =  − 0.552, *P* < 0.001), whereas other factors were not associated with the mean CT in all groups (Table 3).

**Table 3:** Correlation analysis of mean CT and CVI with physiological factors in all groups

	Mean CT		Mean CVI	
	r	*P* Value	r	*P* Value
Age	0.036	0.736	0.060	0.571
Sex	-0.152	0.150	-0.237	0.024
SBP	-0.156	0.139	-0.107	0.315
DBP	-0.187	0.075	-0.215	0.041
Heart rate	-0.150	0.155	-0.174	0.099
Intraocular pressure	-0.060	0.575	-0.158	0.134
BCVA	-0.054	0.610	-0.098	0.357
Posterior staphyloma	-0.280	0.098	-0.237	0.164
Axial length	-0.552	< 0.001	-0.450	< 0.001
Mean CT	*N/A*	*N/A*	0.720	< 0.001

*CT* choroidal thickness, *CVI* choroidal vascularity index, *SBP* systolic blood pressure, *DBP* diastolic blood pressure, *BCVA* best-corrected visual acuity

Correlation analysis demonstrated that axial length was significantly associated with the mean total choroidal area, luminal area, and stromal area in all groups (all *P* < 0.001). Sex, axial length, mean CT and diastolic blood pressure were all related to the mean CVI (Table 3), but multiple linear regression further showed that only mean CT was associated with the mean CVI (R^2^ = 0.523, β = 0.638, *P* < 0.000) in all groups.

The mean choroidal vascular index was not correlated with the presence of posterior staphyloma between the PS group and group 1(Pearson correlation: *r* = -0.237, *P* = 0.164). In PS group, age, sex, heart rate, systolic blood pressure, diastolic blood pressure, intraocular pressure, spherical equivalent dioptres and axial length were all not correlated with mean CT and CVI (all *P* > 0.05), and there was no correlation between mean CT and CVI (*P* > 0.05). Mean PS height was associated with mean CT (Pearson correlation: *r* = -0.578, *P* = 0.039) but not with the mean CVI.

## Discussion

This cross-sectional study aimed to explore the association between pathological myopia and macular choroidal morphology. Posterior staphyloma is the basic lesion of pathological myopia and an indispensable factor in choroidal thinning in high myopia [3]. However, there have been few studies on the relationship between PS and choroidal blood flow in high myopia. The CVI is a reliable non-invasive parameter for evaluating blood flow and vascularity distribution, which can describe the overall condition of the choroid medium and large vessels compared with optical coherence tomography angiography [12]. This study showed a decreasing inclination of the subfoveal CT and CVI in the PS group. In addition, the present study provides new data regarding the relationship between choroidal morphological characteristics and axial length. With the ocular expansion, both the choroidal vessels and stroma decreased.

Our results showed a decrease in CT with axial growth. Potential reasons for choroidal thinning induce atrophy and reduction of choroidal vessels due to axial stretch and loss of retinal pigment epithelium and choriocapillaris. Histological studies show a markedly decreasing distance between the Bruch’s membrane and sclera with axial elongation in myopia, as well as a thinning choroid [28]. This is consistent with previous research conclusions in myopic children and adults [29, 30]. However, some studies believe no association or weak to moderate correlations between CT and axial length [31–33], suggesting that the effects of corneal thickness, lens thickness, sample size, selection bias, and duration of choroidal measurement should be considered [34]. It was found CT was always thinnest nasally, which may be related to the choroidal vascular watershed [35]. Uneven stress distribution at the posterior pole can extract non-uniform reduction of the choroid [3]. In this study, subfoveal thickness seemed more vulnerable than other sectors in axial extension. The foveal area is predicted to be more sensitive to stimulus conditions because it has the highest density of photoreceptors and lower blood perfusion [3, 36].

Owing to mechanical stretching caused by the limited bulge, subfoveal CT significantly decreased in the PS group [3, 16]. However, our study showed that in adults with axial length ≥ 26 mm, a significant reduction was only observed in the superior thickness in the PS subgroup compared with that in group 1. Choroidal thinning is the most common morphological feature at the staphyloma edge, and the edge has the highest incidence in the superior quadrant [15]. Thus, we considered that superior thickness is reduced to a great extent during PS formation and precedes the fovea.

Our study found that the choroidal luminal area and stromal area significantly decreased with axial extension. On the one hand, the stretch of the choroid induced by axial elongation results in decreased density and atrophy of vessels, and axial elongation-associated choroidal thinning affects the Sattler’s and Haller’s layers more markedly than the small vessel layer [37]; on the other hand, the reduction of choroidal stroma in myopia may also be related to the choroidal stretch, but the exact cause has not been reported. Matrix metalloproteases play a role in modulating the synthesis and degradation of the myopic extracellular matrix, causing a thin sclera [38], which has not yet been reported in the myopic choroid. Thus, we predicted that the alteration of choroidal stroma in myopia might be related to matrix metalloproteases, which require further investigation.

The CVI tended to decrease significantly in the longer axial length group compared to the shorter one in the present study, which indicated that despite the decrease in both stroma and vessels during eyeball expansion, the choroidal vessels decreased more than the stroma. This may lead to a decrease in choroidal blood flow and atrophic lesions of the fundus during the progression of myopia. Complete loss of the choriocapillaris and even loss of large choroidal vessels in the atrophic area of eyes with myopic maculopathy observed in angiographic studies [39].

In our study, the CVI in N1, N2, I1 and S2 was lower in the PS group than in group 1. Localized bulging in the PS at the posterior pole can cause mechanical stretching, resulting in thinning of the retina and choroid, and straightening and thinning of the vessels, thereby reducing choroidal capillary perfusion [16, 40]. In addition, the number of choroidal vessels in the PS area decreases [41]. These variations could result in relative ischemia and hypoxia in the posterior pole of the fundus, which may further lead to chorioretinal atrophy or irritate choroidal neovascularization growth. This may be ascribed to the small sample size and lack of participants with choroidal neovascularization or atrophic fundus lesions that showed an insignificant difference in the total choroidal area and the stromal area between the two subgroups. In addition, we found that PS height was the highest in the superior quadrant. The obvious difference in the height of posterior staphyloma in different quadrants may be related to axial elongation and vitreous traction, as well as the impact and direction of gravity [3]. No correlation was found between mean CT, axial length, and PS in this study, and mean PS height was not correlated with AL. This was mainly because of the small number of participants with PS. Ikuno et al. studied thirty-one highly myopic eyes and found that posterior staphyloma height significantly correlated with CT and axial length [33]. After correcting for other factors, we found that only mean CT affected mean CVI.

This study had some limitations. First, only a small number of eyes with PS were included. Second, this hospital-based cross-sectional study may have resulted in a certain amount of bias. Third, we only acquired two-dimensional information about the choroid. In the future, it will be necessary to expand the sample size to study the morphological characteristics of the choroid and changes in its components during eyeball expansion. Three-dimensional imaging can also provide more details of the choroid.

## Conclusion

PS and axial growth were related to the reduction in choroidal stoma and vessels, which induced a thin choroid and choroidal structure remodelling. The degree of choroidal structural changes in the different regions was not consistent. The reduction in the number of vessels was greater than that of the stroma during this process, which may be related to the pathological modification of myopia progression.

## Data Availability

All data generated or analysed during this study are included in this article.

## Abbreviations

PS: Posterior staphyloma
CT: Choroidal thickness
CVI: Choroidal vascularity index
IQR: Interquartile range
